# Scottish and Newcastle Antiemetic Pre-treatment for paracetamol poisoning study (SNAP)

**DOI:** 10.1186/2050-6511-14-20

**Published:** 2013-04-04

**Authors:** H K Ruben Thanacoody, Alasdair Gray, James W Dear, Judy Coyle, Euan A Sandilands, David J Webb, Steff Lewis, Michael Eddleston, Simon HL Thomas, D Nicholas Bateman

**Affiliations:** 1 Wolfson Unit of Clinical Pharmacology, University of Newcastle upon Tyne, & Royal Victoria Infirmary, Newcastle upon Tyne, UK; 2 Emergency Department, Royal Infirmary of Edinburgh, Edinburgh, UK; 3 University/BHF Centre for Cardiovascular Science, Edinburgh University, Edinburgh, UK; 4 NPIS Edinburgh, Royal Infirmary of Edinburgh, Edinburgh, UK; 5 Edinburgh Clinical Trials Unit, University of Edinburgh, Edinburgh, UK

**Keywords:** Paracetamol, Acetylcysteine, Overdose, Antidotes, Hepatotoxicity

## Abstract

**Background:**

Paracetamol (acetaminophen) poisoning remains the commonest cause of acute liver injury in Europe and North America. The intravenous (IV) N-acetylcysteine (NAC) regimen introduced in the 1970s has continued effectively unchanged. This involves 3 different infusion regimens (dose and time) lasting over 20 hours. The same weight-related dose of NAC is used irrespective of paracetamol dose. Complications include frequent nausea and vomiting, anaphylactoid reactions and dosing errors. We designed a randomised controlled study investigating the efficacy of antiemetic pre-treatment (ondansetron) using standard NAC and a modified, shorter, regimen.

**Methods/Design:**

We designed a double-blind trial using a 2 × 2 factorial design involving four parallel groups. Pre-treatment with ondansetron 4 mg IV was compared against placebo on nausea and vomiting following the standard (20.25 h) regimen, or a novel 12 h NAC regimen in paracetamol poisoning. Each delivered 300 mg/kg bodyweight NAC. Randomisation was stratified on: paracetamol dose, perceived risk factors, and time to presentation. The primary outcome was the incidence of nausea and vomiting following NAC. In addition the frequency of anaphylactoid reactions and end of treatment liver function documented. Where clinically necessary further doses of NAC were administered as per standard UK protocols at the end of the first antidote course.

**Discussion:**

This study is primarily designed to test the efficacy of prophylactic anti-emetic therapy with ondansetron, but is the first attempt to formally examine new methods of administering IV NAC in paracetamol overdose. We anticipate, from volunteer studies, that nausea and vomiting will be less frequent with the new NAC regimen. In addition as anaphylactoid response appears related to plasma concentrations of both NAC and paracetamol anaphylactoid reactions should be less likely. This study is not powered to assess the relative efficacy of the two NAC regimens, however it will give useful information to power future studies. As the first formal randomised clinical trial in this patient group in over 30 years this study will also provide information to support further studies in patients in paracetamol overdose, particularly, when linked with modern novel biomarkers of liver damage, patients at different toxicity risk.

**Trial registration:**

EudraCT number 2009-017800-10, ClinicalTrials.gov IdentifierNCT01050270

## Background

Paracetamol (acetaminophen) is the commonest agent taken in overdose in the UK. Paracetamol poisoning had a significant mortality (5–6%) and major morbidity (58%) prior to the introduction of antidotal therapy [1-3]. For example, in a cohort of 57 untreated patients hospitalised with paracetamol overdose, 33 (58%) developed severe hepatocellular injury and 3 (5%) died [2].

After paracetamol overdose, the normal metabolic pathways via sulphation and glucuronidation become saturated, leading to increased formation of the reactive intermediate metabolite, N-acetyl-p-benzoquinoneimine (NAPQI). Small amounts of NAPQI produced during therapeutic paracetamol use can be detoxified safely in glutathione-dependent reactions, but after overdose formation of NAPQI outstrips availability of glutathione, resulting in covalent binding of NAPQI to hepatocytes, culminating in cell death. Thus, without treatment paracetamol overdose can cause hepatocellular injury leading to fulminant hepatic failure and death.

Antidotes for paracetamol poisoning that act by replenishing hepatic glutathione were developed in the 1970s. In the UK, the intravenous N-acetylcysteine (NAC) regimen developed by Prescott and colleagues in Edinburgh has transformed the management of paracetamol poisoning, with liver function abnormalities much less common in treated patients than in untreated historical controls. Despite the complexity of this regimen, involving three different infusions over a 20.25 h period [2], it has been used routinely in the management of paracetamol overdose for over 30 years.

In the USA the lack of an approved NAC preparation for intravenous use led to the development of a 3-day oral NAC regimen [4]. This was used widely and it is only over the past decade there has been a general change to the shorter intravenous NAC regimen in the USA, after its approval by the US Food and Drug Administration (FDA) in 2004.

Neither the oral or intravenous NAC regimens have been subject to formal comparative trials, NAC doses were largely empiric and not subject to more precise dose refinement. NAC dosing is determined on the basis of patient weight for all patients in whom treatment is deemed indicated, irrespective of the ingested dose of paracetamol.

Although highly effective, especially if started within a few hours of paracetamol overdose, the current licensed intravenous NAC regimen has several disadvantages. Firstly, intravenous NAC is commonly associated with adverse effects, the most common being nausea, vomiting and anaphylactoid reactions [5-7]. Anaphylactoid reactions appear related to the rate of infusion of NAC and its concentration in blood. Thus they are most common during, or soon after, the initial high dose infusion. A previous study, however, failed to show that prolonging the initial 15 min infusion to one hour significantly alters their incidence [8]. It is also important to note that anaphylactoid reactions appear more common in patients with lower paracetamol concentrations [7,9,10]. Secondly, the infusion schedule is complex, requiring prescription of three different infusions, and this contributes to a high risk of medication errors [11-13], which may have serious, potentially life-threatening, adverse outcomes [14,15].

Thirdly, the required duration of the infusion regimen results in prolonged hospital stay, especially as patients with paracetamol overdose often present during the night. Since it is difficult to discharge patients after 22.00 and before 08.00, currently any patient presenting between 20:00 and 08:00 is likely to spend a second night in hospital if NAC is administered over 20.25 h and bloods are checked at end of treatment. This effectively means that any patient in whom NAC therapy is commenced is likely to be in hospital well over 24 hours, despite the majority never developing any evidence of liver injury. With a shorter infusion regimen, e.g. over 12 h, patients presenting at any time before 08:00 could potentially be discharged before 22.00, thus reducing the length of hospital stay for a significant proportion of patients. Based on our clinical experience in Edinburgh and Newcastle we estimate that more than 40% of hospital bed occupancy due to paracetamol overdose is due to treatment with NAC. Applying this to the UK, it would amount to 32,000 bed-days/year. We estimate that use of a shorter-duration regimen may potentially save more than 10,000 bed-days per annum across the UK.

We hypothesised that it should be possible to design an alternative method for administering NAC, in which the antidote was delivered using a simpler regimen and for a shorter time, while still providing adequate plasma concentrations of acetylcysteine for liver protection. We approached funders with the view to conducting an initial study of a new modified NAC regimen in patients with paracetamol poisoning. They indicated it would be necessary to do a smaller preliminary study focused on measuring rates of adverse reactions with both regimens, before a larger formal clinical trial of efficacy could be considered. Since no clinical trial had been done in acute paracetamol poisoning in the UK since the 1970s, they also stressed the importance of assessing the practicalities of such a study if a new dose regimen, or possibly even new antidotes, were to be developed. We therefore decided to focus on antiemetic prophylaxis of patients receiving NAC as this is the commonest adverse effect [5-7]. We designed a factorial study in which we compared the antiemetic efficacy of ondansetron with placebo in patients who received standard, or a new modified regimen of NAC. This manuscript describes the methodologies of this study.

## Methods

The study, EudraCT number 2009-017800-10, ClinicalTrials.gov Identifier NCT01050270, was funded by the Scottish Chief Scientist Office (grant no CZB/4/722), approved by the MHRA, the Scotland A Research Ethics Committee, UK (ref no 10/MRE00/20) and sponsored by the University of Edinburgh and NHS Lothian ACCORD Governance & QA Office.

The trial design was developed as a multi-centre study involving 2 acute clinical toxicology units in the Royal Infirmary of Edinburgh and the Royal Victoria Infirmary of Newcastle upon Tyne. A third unit in Aberdeen Royal Infirmary agreed to join the study to achieve adequate patient recruitment within the trial timeframe. The study was primarily designed to test the efficacy of prophylactic antiemetic therapy with ondansetron but was also designed to assess the impact of a new NAC infusion regimen therapy on incidence of adverse effects. A third aim was for the study to provide sufficient experience and data to assess the feasibility of and adequately design and power a study of the modified NAC regimen as a new treatment for this common form of poisoning, as this has a major potential to both reduce patient adverse events from NAC therapy and shorten duration of hospital stay.

### Study design

The trial was conducted using a 2X2 factorial design involving 4 parallel groups with the following treatment allocations (Table 1):

i. Pre-treatment with antiemetic (ondansetron 4 mg IV) compared to matched placebo in a double blind fashion.

ii. Conventional acetylcysteine regimen (300 mg/kg over 20.25 h as: 150 mg/kg in 200 mL over 15 min, then 50 mg/kg in 500 mL over 4 h, then 100 mg/kg in 1000 mL over 16 h) compared to a modified 12 h acetylcysteine regimen (300 mg/kg over 12 h as: 100 mg/kg in 200 mL over 2 h, then 200 mg/kg in 1000 mL over 10 h, followed by 5% glucose over 8 h).

**Table 1:** Drug treatment groups


Ondansetron 4 mg IV Pre-Treatment	Placebo IV Pre-Treatment
Conventional acetylcysteine regimen	Conventional acetylcysteine regimen
Ondansetron 4 mg IV Pre-Treatment	Placebo IV Pre-Treatment
Modified 12 h acetylcysteine regimen	Modified 12 h acetylcysteine regimen

### Rationale for study drugs and doses

i) Ondansetron pre-treatment

It is unclear why no attempt has been made to test antiemetic pre-treatment in the setting of acetylcysteine administration, as only a few other treatments such as cytotoxic chemotherapy are associated with such a high reported rate of nausea and vomiting.

Ondansetron is an antagonist at 5HT3 receptors in the gastrointestinal tract and central nervous system. The licensed indications include prevention of post-operative nausea and vomiting (standard dose 4 mg at induction of anaesthesia) and treatment or prophylaxis of nausea and vomiting with emetogenic cytotoxic chemotherapy (standard dose 8 mg) [16]. It has been shown to be effective in both settings [17] and has a well-characterised adverse effect profile [16]. Common adverse effects include headache, flushing and constipation. Very rarely, hypersensitivity reactions, transient ECG changes (including QT prolongation) and seizures have been reported [16].

Although more expensive than alternative antiemetics such as cyclizine or metoclopramide, ondansetron was chosen for the following reasons:

With regards to the choice of dose, one randomised double-blind dose–response study using six different ondansetron doses (0.5–8 mg) showed that 4 mg was the optimal prophylactic dose and was only barely superior to lower doses [19]. A systematic review of ondansetron in the treatment of PONV also found no differences in efficacy when 1, 4 or 8 mg ondansetron were used [20]. These studies do not support the use of the higher 8 mg dose. The mechanism of nausea and vomiting following paracetamol overdose is not clearly defined but is likely to be multi-factorial, a situation comparable to that of PONV. Nausea and vomiting following paracetamol overdose, although common, is usually not as severe or protracted as after chemotherapy. We, therefore, considered that an antiemetic prophylactic ondansetron dose lower than that used for chemotherapy-induced vomiting and similar to that used for PONV would be appropriate.

For rescue medication the antihistamine cyclizine was chosen as this was significantly better than a repeat 4 mg dose of ondansetron (complete response 78% vs. 46%) in PONV [21]. Also, in a randomised clinical trial of repeat 4 mg ondansetron dose rescue therapy vs. placebo, ondansetron was no better than placebo at controlling PONV [22]. Based on these studies in PONV, we chose an initial dose of 4 mg ondansetron, which is the licensed and effective dose of ondansetron for PONV with intravenous cyclizine 50 mg used as rescue therapy for those with continuing symptoms.

i) it is the most frequently used licensed drug for the prophylactic treatment of post-operative nausea and vomiting (PONV) and chemotherapy-induced vomiting;

ii) there is considerably more randomised controlled trial evidence supporting ondansetron for this prophylactic use compared to other older and less expensive alternatives [18];

iii) anti-dopaminergic agents such as metoclopramide may cause dystonic reactions, particularly in the younger age group likely to be represented in this trial;

iv) cyclizine has anti-histaminergic activity. Since one of the aims of this study was to examine the effect of the modified NAC regimen on rates of histamine-mediated anaphylactoid reactions it was felt that the use of cyclizine could have led to an interaction between the antiemetic and the rates or severity of anaphylactoid reactions in this study. This would thus make power calculations for future studies more difficult.

ii) Acetylcysteine infusion regimen

**Figure 1:**
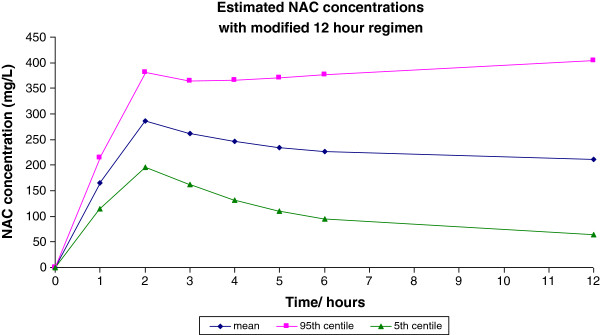
Monte Carlo calculations of proposed modified acetylcysteine regimen.

We consider that if it were possible to use a 12 h infusion of NAC it could significantly reduce hospital stay. There is also evidence suggesting that high concentrations of NAC following the initial loading infusion in the Prescott regimen are likely related to the incidence of adverse drug reactions (ADRs) [7]. Using pharmacokinetic data derived from Prescott [23], we performed a Monte Carlo simulation to derive expected plasma concentrations, based on a 1-compartment model, using a new shorter 300 mg/kg NAC regimen. This consisted of a two hour infusion of 100 mg/kg followed by ten hours of 200 mg/kg, both in 5% dextrose. Applying this we estimated that the modified infusion regimen would give rise to a mean plasma concentrations of 306, 122 and 30 mg/L at 2 h, 12 h and 20 h respectively and that the probability that the peak plasma acetylcysteine concentration at the end of the 2 h loading infusion would be greater than 150 mg/L and less than 650 mg/L was greater than 99%. This regimen would therefore administer the same total dose of NAC but achieve a lower peak and a 20 h plasma concentration comparable to the conventional regimen (Figure 1) [23]. In preliminary studies in healthy volunteers examining the effects of the modified NAC regimen on renal function, plasma concentration data was in line with these predictions [24].

Assuming that anaphylactoid reactions to acetylcysteine are concentration-related and that this occurs in patients who achieve a peak concentration more than 1 standard deviation above the mean (based on the observation that about 15% of patients develop such reactions [8], the pharmacokinetic data with the conventional regimen suggest that these reactions occur at concentrations of greater than 650 mg/L. Simulated data using the proposed modified regimen suggest that <1% of patients would achieve an NAC concentration of >650 mg/L, therefore, potentially leading to a marked reduction in the incidence of anaphylactoid reactions. Preliminary data from healthy volunteers and patients in a clinical trial using the new NAC regimen as prophylaxis against contrast-induced liver injury, has found that nausea or vomiting have not occurred [24], suggesting that acute adverse effects will be less common with this new regimen in patients who receive it for paracetamol poisoning.

In order to approximately match fluid regimens between conventional and modified regimens, the modified regimen included a terminal 8 h 5% dextrose infusion (Table 1).

#### Identifying patients

Potentially eligible patients were identified by treating clinicians at the Emergency Departments of the Royal Infirmary of Edinburgh; the Medical Assessment Unit and the Emergency Department the Royal Victoria Infirmary, Newcastle; and the Emergency Department of Aberdeen Royal Infirmary. A trained member of staff then assessed the patient for study eligibility.

Patients were deemed eligible for consideration if they presented to the relevant acute units within 36 hours of a single acute paracetamol overdose and required treatment with NAC, based on standard UK guidance for management as published on TOXBASE and in the British National Formulary. The decision to treat depends on the paracetamol concentration in relation to the time since ingestion and is interpreted using a treatment nomogram. Until Sept 2012 treatment was indicated for patients without additional risk factors for paracetamol hepatotoxicity presenting with a timed paracetamol concentration above a line commencing at 200 mg/L at 4 h after overdose and falling exponentially with a half-life of 4 h (the ‘200’ line). For patients with at least one risk factor, treatment was indicated at concentrations 50% lower than this, i.e. above a ‘100’ treatment line [25]. Risk factors were defined as follows in two categories:

a) Nutritional deficiency, malnourished and/or debilitating disease: acute or chronic starvation, eating disorders, cachexia, malabsorption syndromes, AIDS, cystic fibrosis, hepatitis C, chronic alcoholism.

b) Enzyme induction: use of drugs with this property (carbamazepine, rifampicin, barbiturates, phenytoin, rifabutin, efavirenz, nevirapine, St John’s Wort); regular consumption of ethanol above advised amounts.

For patients presenting more than eight hours after ingestion, and at risk of liver damage based on history of dose ingested, treatment with acetylcysteine is started immediately pending the results of plasma paracetamol concentration measurement. For those presenting within 8 h, current recommendations are to wait for results of plasma paracetamol concentration measurement before starting NAC. After Sept 2012 the trial was modified in line with new UK guidance on management of paracetamol poisoning (see below).

#### Trial exclusions

The following exclusion criteria were applied throughout the trial:

patients less than 16 years old; those detained under the Mental Health Act; those with known permanent cognitive impairment; patients with a life-threatening illness; patients known to be pregnant; those who had previously participated in the study; those considered to have an unreliable history of paracetamol overdose; patients presenting more than 36 hours after overdose (24 h up to May 2011) of a single acute paracetamol overdose; patients presenting after taking a staggered paracetamol overdose (defined as when the overdose of paracetamol was taken over a period of more than 2 h (1 h up May 2011); patients taking anticoagulants (e.g. warfarin) in therapeutic doses or in overdose; patients who, in the opinion of the responsible clinician/nurse, were unlikely to complete the full course of NAC e.g. expressing wish to self-discharge: patients who in the opinion of the responsible clinician/nurse were unable to complete the initial questionnaire either themselves or with nurse assistance; a history of hypersensitivity to 5HT3 antagonists; non-English speaking patients. Trial information material was only produced in English in view of the known and stable demographic of the Edinburgh, Newcastle and Aberdeen self harm population.

#### Data collection

The following data were collected at baseline prior to randomisation: Patient demographics (age and gender); height and weight; screening and eligibility criteria; paracetamol poisoning information including risk assessment; co-ingestion of other drugs or ethanol. This information was collected directly onto a patient recruitment and randomisation forms, and undertaken by delegated trained clinical or research staff.

Consent was taken prior to randomization by delegated trained clinical staff and recorded on a consent form in triplicate, with one copy given to the participant; one placed in the research files and one kept in the hospital notes.

Study treatment information was collected during the treatment phase and recorded on the Case Report Form by trained clinical or research staff. Adverse reactions (at baseline, 2 h and 12 h) - were measured via an 11-point Likert scale (whereby 0 = none, 10 = very severe) on the following 9 symptoms. This was a self-assessment done by the patient (or completed by the nurse if the patient was physically unable to complete the form) and completed as a questionnaire.

• Nausea

• Feeling flushed

• Itchy skin

• Skin rash

• Chest pain

• Headache

• Feeling breathless

• Feeling wheezy

• Tongue/lip swelling

In addition episodes of vomiting and retching were recorded objectively by nursing staff at baseline, and at 2 h and 12 h after initiation of acetylcysteine.

Blood samples for paracetamol concentration, INR, urea and electrolytes (U&Es), creatinine, full blood count (FBC) and liver function tests including ALT, bilirubin and gamma glutamyl transferase (GGT) were obtained at baseline and at 12 h and 20.25 h after initiation of acetylcysteine. These samples were taken by clinical staff. Results were collected by the research team, via the hospital electronic laboratory reporting system.

Blood pressure and pulse were recorded at the following time points: 0 h, 15 min, 2 h, 4 h, 12 h and 20.25 h (end of treatment).

Any use of rescue medication was recorded contemporaneously in the Case Report Form by clinical staff. Any other clinical features were also collected from the clinical records.

#### Novel biomarker substudy

Each time a blood sample was taken in the main study or pharmacokinetic sub study, 2 additional samples were obtained for the measurement of the proteomic and inflammatory response to paracetamol poisoning.

#### Pharmacokinetic substudy

Acetylcysteine concentrations were measured in a convenience sample of patients in Edinburgh. It was agreed that samples could be obtained at any of the potential time points in consenting patients; baseline, 15 mins, 2 h, 4 h or 12 h and end of treatment. These samples were taken by nominated research staff and stored for subsequent analysis.

#### Discharge

Outcome and survival data were collected by the research team via the hospital electronic system and clinical notes, and recorded in the Case Report Form.

#### Adverse events

These were monitored and recorded contemporaneously in the Case Report Form by clinical staff, consistent with the sponsor’s quality and assurance processes. These were monitored as agreed by a Data Monitoring Committee comprising Prof RE Ferner (Clinical Pharmacologist, Birmingham, Chair), Dr K Simpson (Hepatologist, Edinburgh) and Dr J E Gray (Statistician, Leeds).

#### Data for analysis

All data was entered onto a purpose built web-based database to be analysed within the Edinburgh Clinical Trials Unit.

### Consent process

The trial was undertaken in accordance with the Medicine for Human Use (Clinical Trials) Regulations 2004 and all subsequent amendments. The main ethical challenge was that potential participants were acutely ill and may initially have lacked capacity to provide informed consent, or the ability to complete a written consent form, yet the very nature of the trial required that recruitment took place quickly in an emergency and included acutely ill patients.

Patients (and if present and appropriate their accompanying relative) were provided with a Summary Patient Information Sheet, and given time to consider the trial and ask questions regarding their participation. They were interviewed by a delegated recruiting doctor, middle grade or above, who had completed GCP training, including specific training in assessing capacity. Potential participants were given further verbal information before being asked to provide written witnessed consent.

Potential participants who were able to express consent, but unable to complete the consent form, were recorded as having provided verbal witnessed consent. Subsequent written consent for continuation in the trial was sought as soon as possible after recruitment. In the event of a patient refusing consent at this point, the patient was withdrawn and no further data collected, but the patient’s permission was sought to use the data collected up to that point. If the patient objected to this, all data collected for the patient was destroyed. All patients were, however, strongly advised to complete their treatment in order to protect their liver from paracetamol toxicity. As soon as the patient’s condition allowed they were provided with the Full Patient Information sheet (as soon as possible after the initial emergency has passed).

**Figure 2:**
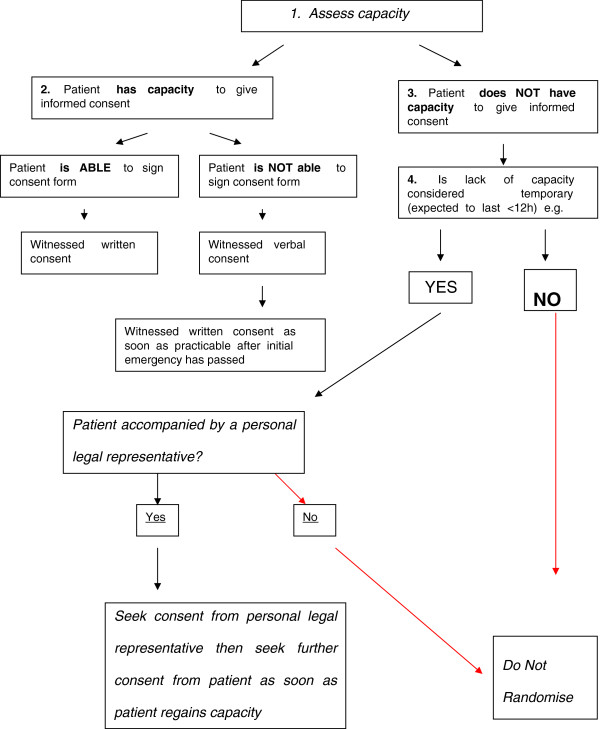
Consent flowchart.

While a majority of patients were able to provide witnessed written or verbal consent, a minority lacked initial capacity to consent because of reduced conscious level or cognitive impairment. In those where loss of capacity was deemed to be temporary e.g. alcohol or drug intoxication, consent was sought after discussion and agreement from a relative Figure 2.

### Randomisation process

Randomisation was performed via a web-based randomisation service, managed by the Edinburgh Clinical Trials Unit, after a delegated recruiting doctor had assessed a subject as meeting the eligibility criteria. Baseline data entered on to the system were stored securely and the treatment allocation was provided. Once a patient was randomised he/she remained in the study and had all outcomes recorded regardless of compliance with medication, unless he/she specifically withdrew consent to have data stored.

A minimisation program, including a random element, was used to achieve optimum balance for the most important prognostic factors:

• Time from ingestion to treatment dichotomised as <8 h and 8–24 (later 36) h.

• Presence of any, vs. absence of all of the risk factors.

• Reported paracetamol dose, dichotomised as <16 g and >16 g.

This minimisation program was applied independently at all recruitment sites to try to ensure balance across sites. It was not changed after the MHRA advice of September 2012, which reduced threshold for treatment to the “100 mg” line, although it was expected that a consequently higher proportion of “low risk” patients would be recruited in the final weeks of the trial.

All participants, the medical and nursing staff caring for them and the research teams were blinded to the antiemetic treatment allocation. Patient packs, specially prepared and packaged for clinical trial purposes, containing closely matched ondansetron (2 mg/mL) and placebo (0.9% saline) 2 mL ampoules were prepared and labelled to patient allocation number to reduce the chance of code break. Stores of packs were held within the hospital pharmacy and sufficient packs supplied as required.

Doses of NAC were based on the patient’s weight and therefore infusions need to be prepared individually for each patient. As a result it was not feasible for patients, clinicians or researchers to be blinded to treatment allocation. The infusions were designed to run over 20.25 h in both treatment arms to make the treatment allocation less obvious and patients were not told which treatment they have been assigned to.

Treatment allocation was on a 1:1:1:1 ratio across the 4 parallel drug treatment groups.

### Substantial amendments to trial protocol

The trial commenced in September 2010 and since that date 2 major protocol amendments have been made.

i) Recruitment was initially extremely slow and we identified definition of “staggered” overdose as the reason for this. The initial study defined a “staggered” overdose as ingestion over 1 h or more. We soon became aware that in reality a substantial proportion of patients consumed their tablets over a longer period than 1 h.

The protocol was therefore amended after discussion between the investigators, the DMC, the Ethics Committee and the Sponsor. From May 2011 eligibility criteria changed to include patients who had taken an overdose over a period of less than 2 h (previously 1 h). In addition the maximum time after overdose that patients could be included in the trial was increased from 24 to 36 h.

ii) A second more radical change occurred in September 2012, when on the advice of the UK Commission on Human Medicines (CHM) the MHRA published new guidelines for the management of acute paracetamol poisoning. These recommended use of a single ‘100’ nomogram treatment line for all patients. They also recommended that risk factors should no longer be taken into account when considering the need for acetylcysteine. After discussion with the investigators, MHRA clinical trials unit, the Ethics Committee and the Trial Data Monitoring Group this new treatment limit was adopted in the trial as from 6th Sep 2012. We did not, however, change our approach to dosing with NAC, in particular there was no change in the rate of infusion in the standard therapy group, although CHM and MHRA had recommended a longer (1 h) duration for the initial 150 mg/kg acetylcysteine infusion.

### Events and outcomes

#### Primary objective

To determine whether pre-treatment with intravenous ondansetron 4 mg reduces the occurrence of retching or vomiting in paracetamol poisoned patients receiving intravenous acetylcysteine. Retching was defined as an attempt to vomit not producing any liquid. The primary endpoint was the proportion of patients in each group who do not vomit or retch within 2 hours of initiation of NAC treatment and had no use of rescue medication.

#### Secondary objectives

1) To determine whether pre-treatment with intravenous ondansetron 4 mg will reduce the occurrence of nausea in paracetamol poisoned patients receiving intravenous NAC within 12 h of initiation of that treatment. Nausea severity was assessed using an 11–point, whole number, categorical Likert scale, with 0 representing “no nausea” and 10 representing nausea “as bad as it can possibly be” [26,27]. Patients were deemed to have failed to have adequate control of nausea if they had a score of greater than 4 at any time up to 12 h after commencement of NAC or if they received rescue medication [27].

Additional data collected will inform the design of future clinical studies, although this study is not powered to detect differences in efficacy or safety between the two NAC infusion regimens:

2) To compare the incidence of anaphylactoid reactions in the modified and conventional NAC regimens in paracetamol poisoned patients. This is to be derived from the case records, need for treatment of an anaphylactoid response and self-reported incidence of flushing, itchy skin, skin rash, chest pain, feeling breathless, feeling wheezy and tongue/lip swelling on the Likert scales and documented changes in blood pressure and pulse rate.

Symptom data is collected on 11-point Likert scales with upper and lower descriptors of “none” and “very severe” at baseline, 2 and 12 h. A score of 5 or over will be taken to represent a positive feature on each scale. The domains the scales cover are as follows:

Skin features

• Feeling flushed

• Itchy skin

• Skin rash

Respiratory

• Feeling breathless

• Feeling wheezy

Systemic features

• Tongue/lip swelling

• Chest pain

• Change in BP and pulse

A positive score within a domain of skin, respiratory or systemic, will be used to indicate a response in the grading assessment of anaphylactoid response described below. A fall in systolic pressure of 20 mmHg or more, or rise in pulse rate of 20 bpm or more will be taken as positive features of an anaphylactoid response.

In addition we will have access to data on blood pressure and pulse rate change at 15 min, 4 hours post commencement of infusion, and these are the times when anaphylactoid responses to NAC are most likely.

Anaphylactoid reaction severity will be assessed on a 3-grade severity scale, based on the World Allergy Organization guidelines (2011) [28].

*Grade 1*. The least severe group would be patients who only had a positive response in one of the domains on the Likert scales, or change in blood pressure or pulse rate as defined above.

*Grade 2*. The intermediate grading would be patients who had positive responses in 2 or more symptom domains on the Likert scales, and/or cardiovascular change measured in blood pressure or pulse, but did not require cessation of NAC therapy, or other specific treatments.

*Grade 3*. The most severe grading would be patients who had NAC treatment discontinued and/or an intervention with anti-allergy therapy.

3) To determine the rate of occurrence of hepatotoxicity in patients treated overall and with the modified and conventional NAC administration regimens. These to be determined as:

a) Proportion of patients with a 50% increase in ALT after 20.25 h post-treatment with NAC, compared with the admission value overall, in each treatment arm, and in those receiving the different NAC regimens.

b) Proportion of patients with ALT > 1000 at 20.25 h post-treatment with NAC overall, in each treatment arm, and in those receiving the different NAC regimens.

c) Proportion of patients with INR > 1.3 at 20.25 h post-treatment with NAC overall, in each treatment arm, and in those receiving the different NAC regimens.

4) To measure total length of hospital stay in patients receiving modified and conventional acetylcysteine treatment regimens.

5) To identify new blood markers for paracetamol-induced liver injury. These included protein and RNA markers during both conventional and modified regimens, in order to find new markers that identify organ injury earlier in the disease process than current markers [29]. This is critical for further development of the modified NAC regimen as patient stratification and early rule out of injury are essential as a companion diagnostic.

6) In addition a convenience subsidiary sample of patients were collected in a trial subset in Edinburgh, and had blood samples taken to measure plasma acetylcysteine concentrations of modified and conventional regimens.

### Statistical analysis

In a Cochrane Database systematic review of antiemetics used in the prophylaxis of PONV, treatment with prophylactic ondansetron compared to no drug treatment was associated with a risk ratio of 0.42 (95% CI 0.34–0.52) for vomiting and a risk ratio of 0.56 (95% CI 0.44–0.72) for nausea or vomiting.

To achieve at least 80% power to detect a relative risk of 0.6 for the proportion of patients with retching or vomiting within 2 hours (from 60% in the treated group to 36% in the placebo group), 91 patients need to be enrolled in each group (significance level set at 0.25 to allow for 2 comparisons in factorial study). To allow for a potentially higher drop-outs/non-compliance rate in this challenging population, in whom a proportion would be randomized without paracetamol concentration data available (those >8 h post overdose), we planned to include 250 patients, 125 patients randomised to ondansetron, and 125 to placebo. This was to ensure 50 patients in each of the four groups in the factorial study (ondansetron with conventional regimen, placebo with conventional regimen, ondansetron with modified regimen and placebo with modified regimen), and allow for a 20% trial drop out in the analysis of the primary outcome.

The trial will be analysed for all outcomes on an intention-to-treat basis, although because of the trial design, and need to recruit late presenting patients prior to paracetamol concentration data being available, some may not achieve the primary endpoint. These patients therefore cannot be included in an end-point analysis.

## Discussion

Paracetamol is the commonest drug taken in overdose in the United Kingdom, including in Scotland [30-32]. Hospitalisation due to paracetamol overdose accounted for approximately 80,000 bed days in the UK in 2005–2006 (source: Hospital Episode Statistics & ISD Scotland). A substantial proportion of this time results from use of NAC. Although highly effective in preventing liver damage if given sufficiently early, further disadvantages of the conventional intravenous NAC infusion regimen are the high rates of adverse reactions and the complexity of dosing and resulting high risk of medication errors.

To address these disadvantages, a modified NAC dose regimen has been designed to be shorter and simpler than the conventional regimen and associated with lower initial plasma concentrations in an attempt to reduce the incidence of dose-related adverse effects. This modified regimen administers the same total dose in 2 separate infusions instead of 3 and, therefore, may reduce the risks of error associated with preparing infusions. Ideally, such a regimen would be used in routine clinical practice after an adequately powered efficacy study had been performed. In theory, the slower initial delivery of NAC may result in less effective early generation of hepatic glutathione for detoxification of NAPQI, although any difference between the current 1 h and proposed and 2 h regime is likely to be small. Also, the shorter total duration of the infusion may mean that delivery of NAC ceases before all NAPQI has been formed and detoxified, leaving some patients inadequately treated. Such patients would be expected to have detectable paracetamol concentrations at the end of the 12 h infusion and the frequency of this finding is being assessed in this clinical trial.

Ideally, any efficacy study comparing new and conventional acetylcysteine regimens would need to be powered to establish non-inferiority. Based on the efficacy of current NAC treatment, however, very large numbers of participants would be required. Nevertheless a safer infusion causing fewer severe ADRs would also benefit patients and health care workers, freeing their time from managing the high frequency ADRs associated with paracetamol overdose and its treatment.

Design and conduct of this randomised controlled trial was associated with a number of problems, both ethical and practical. Patients with self-harm are a potentially challenging group to recruit into a clinical trial. We had undertaken a survey of our patients with paracetamol poisoning in Edinburgh prior to undertaking the study in order to assess their views on being approached for consent, and the need for additional blood sampling. Many of these patients had received NAC in the past and were therefore aware of the adverse effects caused. Patients being treated with acetylcysteine in Edinburgh were approached to seek their views about whether they would wish to take part in a clinical trial involving the modified acetylcysteine regime. They were given a 1-page summary of the research, asked about their willingness to participate and their views about consent. Patients overwhelmingly thought the research would be useful and they would be willing to take part. With regards to consenting procedures, if they were unable to give informed consent, most preferred that, if a relative did not accompany them, the decision to involve them in the study were taken by the treating clinician. We therefore adopted the policy that ethics consent would not be sought from relatives by telephone, but only from relatives accompanying the patient and therefore already aware of their presentation. We also considered the use of an alternative personal legal representative, proposing the senior nurse in the hospital, but the ethics committee felt this was also unacceptable. This resulted in a reduction in the number of potential recruits into the study.

As detailed in the section on study amendments, we originally believed that most patients would take paracetamol overdose over a period of less than an hour. It quickly became clear that many of our patients, even though they took their tablets at one sitting, took these over longer than the hour originally specified in the trial documentation. We believe this is a novel finding and may explain some of the difficulties reported in using paracetamol nomograms, particularly if blood samples are taken around four hours after commencement of drug ingestion. The ethics committee accepted a change in overall ingestion time to less than two hours as representing the time that would be used for including patients within the trial. We also extended the inclusion time to 36 h from ingestion to fall in line with national advice provided by the UK National Poisons Information Service.

Assessing severity of anaphylactoid reactions will be based on the 5-point scale proposed by the World Allergy Association (WAA) [28]. In this scale the most severe reaction (Grade 5) is death. Although rarely reported, death is extremely unlikely with NAC in this study. Gastrointestinal features, including nausea, are also included by the WAA, and it is inappropriate to include this in an antiemetic efficacy study of the current design. We have therefore adopted a 3-grade study assessment based on the WAA categories, and using organ domains as the basis of the severity grading.

Recruitment targets were necessarily set higher than required for the actual analysis since we had no data to judge drop-out rates and adopted a 20% overall drop-out as a potential worst case scenario when calculating trial target recruitment. Sri Lankan experience suggested actual drop-out rates might be much lower, and this aspect is one that will inform any future studies in this patient population [33].

If a shorter acetylcysteine regimen is to be used clinically it will be important to establish appropriate markers to reliably inform early patient discharge. Some have been suggested [34] but none has been tested prospectively. In the present study we are addressing this using conventional blood tests taken after 12 h of NAC therapy in both treatment arms, as well as measuring these same end-points at the time of end of conventional therapy (20.25 h) in all treatment groups. By using novel markers of hepatotoxicity [29] we anticipate it may be possible to discharge patients safely after the 12 h modified regimen, but this will need testing in a properly powered study. Our trial should provide the data to allow such a power calculation to be accurately performed.

## Conclusion

In conclusion this study is the first attempt to undertake a formal clinical trial in paracetamol poisoning in the UK since the 1970s. Developing clinical trial evidence in the population of self-harm patients is important since at present much of the management of poisoned patients is based on anecdote. These studies are potentially difficult, and may involve very large numbers of patients [33], but are essential if we are to make advances in the care of this large patient population. Taken with the potential for new markers that could give earlier indications of toxicity, or lack of it, the novel approach to giving NAC appears to offer the potential for a new dimension to managing paracetamol overdose in future.

## Competing interests

The authors declare that they have no competing interests.

## Authors’ contributions

HKRT and DNB originated the design of this study and wrote the grant application. ME, SHLT, AG, JWD, DJW and EAS all contributed to the design and the running of the trial. SL provided statistical advice and JC was the trial manager. All authors other than SL contributed to the day to day running of the study. All authors read and approved the final manuscript.

## Pre-publication history

The pre-publication history for this paper can be accessed here:

http://www.biomedcentral.com/2050-6511/14/20/prepub

## References

[B1] Prescott LF Illingworth RN Critchley JA Stewart MJ Adam RD Proudfoot AT Intravenous N-Acetylcysteine: the treatment of choice for paracetamol poisoning BMJ 1979 ii 1097 1100 519312 10.1136/bmj.2.6198.1097 PMC1597048

[B2] Prescott LF Ballantyne A Park J Adriaenssens P Proudfoot AT Treatment of paracetamol (acetaminophen) poisoning with N-acetylcysteine Lancet 1977 432 434 70646 10.1016/s0140-6736(77)90612-2

[B3] Brok J Buckley NA Gluud C Interventions for paracetamol (acetaminophen) overdose Cochrane Database Syst Rev 2006 Art. No. 2 CD003328 16625578 10.1002/14651858.CD003328.pub2

[B4] Smilkstein MJ Knapp JL Kulig KW Rumack BH Efficacy of oral N-acetylcysteine in the treatment of acetaminophen overdose. Analysis of the national multicenter study (1976 to 1985) N Engl J Med 1988 319 1557 1562 10.1056/NEJM198812153192401 3059186

[B5] Lynch RM Robertson R Anaphylactoid reactions to intravenous N-acetylcysteine: a prospective case controlled study Accid Emerg Nurs 2004 12 10 15 10.1016/j.aaen.2003.07.001 14700565

[B6] Mullins ME Schmidt RU Jr Jang TB What is the rate of adverse events with intravenous versus oral NAC in pediatric patients? Ann Emerg Med 2004 44 547 549 10.1016/j.annemergmed.2004.03.051 15523751

[B7] Pakravan N Waring WS Bateman DN Risk factors and mechanisms of anaphylactoid reactions to acetylcysteine in acetaminophen overdose Clin Toxicol 2008 46 697 702 10.1080/15563650802245497 18803085

[B8] Kerr F Dawson A Whyte IM Buckley N Murray L Graudins A Chan B Trudinger B The Australasian Clinical Toxicology Investigators Collaboration randomized trial of different loading infusion rates of N-acetylcysteine Ann Emerg Med 2005 45 402 408 10.1016/j.annemergmed.2004.08.040 15795719

[B9] Schmidt LE Dalhoff K Risk factors in the development of adverse reactions to NAC in patients with paracetamol poisoning Br J Clin Pharmacol 2001 51 87 91 11167669 10.1046/j.1365-2125.2001.01305.x PMC2014432

[B10] Waring WS Pettie JM Dow MA Bateman DN Paracetamol appears to protect against N-acetylcysteine-induced anaphylactoid reactions Clin Toxicol 2006 44 441 442

[B11] Ferner RE Langford NJ Anton C Hutchings A Bateman DN Routledge PA Random and systematic medication errors in routine clinical practice: a multicentre study of infusions using acetylcysteine as an example Br J Clin Pharmacol 2001 52 573 577 10.1046/j.0306-5251.2001.01490.x 11736866 PMC2014610

[B12] Hayes BD Klein-Schwartz W Doyon S Frequency of medication errors with 21-hour acetylcysteine for acetaminophen overdose Ann Pharmacother 2008 42 766 770 10.1345/aph.1K685 18445707

[B13] Fathalla S Hill S Hodson K Salih IS Thomas SHL Inaccuracies in acetylcysteine dose calculation or infusion rates in patients with and without anaphylactoid reactions Clin Toxicol 2008 46 401 402

[B14] Bailey B Blais R Letarte A Status epilepticus after a massive intravenous N-acetylcysteine overdose leading to intracranial hypertension and death Ann Emerg Med 2004 44 401 406 10.1016/j.annemergmed.2004.05.014 15459624

[B15] Little M Murray L McCoubrie D Daly FF A potentially fatal prescribing error in the treatment of paracetamol poisoning Med J Aust 2005 183 535 536 16296969 10.5694/j.1326-5377.2005.tb07157.x

[B16] SPC Zofranhttp://www.medicines.org.uk/emc/medicine/17650/spc (accessed 02/01/2013)

[B17] Markham A Sorkin EM Ondansetron: an update of its therapeutic use in chemotherapy-induced and postoperative nausea and vomiting Drugs 1993 45 931 952 10.2165/00003495-199345060-00006 7691500

[B18] Carlisle J Stevenson CA Drugs for preventing postoperative nausea and vomiting Cochrane Database Syst Rev 2006 3 Art. No.: CD004125 10.1002/14651858.CD004125.pub2 PMC6463839 16856030

[B19] Dershwitz M Conant JA Chang Y Rosow CE Connors PM A randomized, double-blind, dose–response study of ondansetron in the prevention of postoperative nausea and vomiting J Clin Anesth 1998 10 314 320 10.1016/S0952-8180(98)00035-X 9667348

[B20] Tramèr MR Moore RA Reynolds DJM McQuay HJ A quantitative systematic review of ondansetron in treatment of established postoperative nausea and vomiting BMJ 1997 314 1088 1092 10.1136/bmj.314.7087.1088 9133892 PMC2126477

[B21] Habib AS Gan TJ The effectiveness of rescue antiemetics after failure of prophylaxis with ondansetron or droperidol: a preliminary report J Clin Anesth 2005 17 62 65 10.1016/j.jclinane.2004.04.004 15721732

[B22] Kovac AL O'Connor TA Pearman MH Kekoler LJ Edmondson D Baughman VL Angel JJ Campbell C Jense HG Mingus M Shahvari MB Creed MR Efficacy of repeat intravenous dosing of ondansetron in controlling postoperative nausea and vomiting: a randomized, double-blind, placebo-controlled multicenter trial J Clin Anesth 1999 11 453 459 10.1016/S0952-8180(99)00067-7 10526822

[B23] Prescott LF Donovan JW Jarvie DR Proudfoot AT The disposition and kinetics of intravenous N-acetylcysteine in patients with paracetamol overdosage Eur J Clin Pharmacol 1989 37 501 506 10.1007/BF00558131 2598989

[B24] Eddleston M Megson I Sandilands E 2012 unpublished

[B25] British National Formulary. 58th Edition 2009 BMJ Group and RPS publishing

[B26] White PF Tang J Hamza MA Ogunnaike B LO M Wender RH Naruse R Sloninsky A Kariger R Cunnean S Khalili T The use of oral granisetron versus intravenous ondansetron for antiemetic prophylaxis in patients undergoing laparoscopic surgery: the effect on emetic symptoms and quality of recovery Anesth Analg 2006 102 1387 1393 10.1213/01.ane.0000208967.94601.cd 16632815

[B27] Diemunsch P Gan TJ Philip BK Girao MJ Ederhart L Irwin MG Pueyo J Chelly JE Carides AD Reiss T Evans JK Lawson FC for the Aprepitant-PONV Protocol 091 International Study Group Single-dose aprepitant vs ondansetron for the prevention of postoperative nausea and vomiting: a randomized, double-blind Phase III trial in patients undergoing open abdominal surgery Br J Anaesthesia 2007 99 202 211 10.1093/bja/aem133 17540667

[B28] World Allergy Organization guidelines for the assessment and management of anaphylaxis http://www.espai-eg.org/WAO%20anaphylaxis%20guidelines_WAOJ%202011.pdf (accessed Dec18 2012)

[B29] Antoine DJ Jenkins RE Dear JW Williams DP McGill MR Sharpe MR Craig DG Simpson KJ Jaeschke H Park BK Molecular forms of HMGB1 and Keratin-18 as mechanistic biomarkers for mode of cell death and prognosis during clinical acetaminophen hepatotoxicity J Hepatol 2012 56 1070 1079 10.1016/j.jhep.2011.12.019 22266604 PMC4127883

[B30] Camidge DR Wood RJ Bateman DN The epidemiology of self-poisoning in the UK Br J Clin Pharmacol 2003 56 613 619 10.1046/j.1365-2125.2003.01910.x 14616420 PMC1884308

[B31] Bateman DN Bain M Gorman D Murphy D Changes in paracetamol, antidepressants and opioid poisoning in Scotland during the 1990s Q J Med 2003 96 125 132 10.1093/qjmed/hcg015 12589010

[B32] Ferner RE Dear JW Bateman DN Management of paracetamol poisoning BMJ 2011 342 d2218 10.1136/bmj.d2218 21508044

[B33] Eddleston M Juszczak E Buckley NA Senarathna L Mohamed F Dissanayake W Hittarage A Azher S Jeganathan K Jayamanne S Sheriff MHR Warrell DA for the Ox-Col Poisoning Study collaborators Multiple-dose activated charcoal in acute self-poisoning: a randomised controlled trial Lancet 2008 371 579 587 10.1016/S0140-6736(08)60270-6 18280328 PMC2430417

[B34] Sivilotti ML Yarema MC Juurlink DN Good AM Johnson DW A risk quantification instrument for acute acetaminophen overdose treated with N-Acetylcysteine Ann Emerg Med 2005 46 263 271 10.1016/j.annemergmed.2005.04.004 16126138

